# Identification of Chromosome Abnormalities in Subtelomeric Regions Using Multiplex Ligation Dependent Probe Amplification (MLPA) Technique in 100 Iranian Patients With Idiopathic Mental Retardation

**DOI:** 10.5812/ircmj.8221

**Published:** 2013-10-05

**Authors:** Farkhondeh Behjati, Saghar Ghasemi Firouzabadi, Firoozeh Sajedi, Kimia Kahrizi, Mostafa Najafi, Behruz Ebrahimizade Ghasemlou, Yousef Shafeghati, Fatemeh Behnia, Ali Reza Mohammadi Arya, Hossein Karimi, Fatemeh Hadipour, Zahra Hadipour, Peyman Jamali, Roxana Kariminejad, Hossein Darvish, Ideh Bahman, Eiman Bagherizadeh, Hossein Najmabadi, Roshanak Vameghi

**Affiliations:** 1 Genetics Research Center, University of Social Welfare and Rehabilitation Sciences, Tehran, IR Iran; 2 Pediatric Neurorehabilitation Research Center, University of Social Welfare and Rehabilitation Sciences, Tehran, IR Iran; 3 Department of Occupational Therapy, University of Social Welfare and Rehabilitation Sciences, Tehran, IR Iran; 4 University of Social Welfare and Rehabilitation Sciences, Tehran, IR Iran; 5 Zafar Rehabilitation Clinic, Tehran, IR Iran; 6 Sarem Cell Research Center, Sarem Hospital, Tehran, IR Iran; 7 Shahroud Welfare Organization, Shahroud, IR Iran; 8 Pathology and Genetics Center, Tehran, IR Iran; 9 Department of Medical Genetics, Shahid Beheshti University of Medical Sciences, Tehran, IR Iran

**Keywords:** Ligation, Mental Retardation, Hypersomnolence Idiopathic

## Abstract

**Background:**

Mental retardation/Developmental delay (MR/DD) is present in 1 - 3% of the general population (1, 2). MR is defined as a significant impairment of both cognitive (IQ < 70) and social adaptive functions, with onset before 18 years of age.

**Objectives:**

The purpose was to determine the results of subtelomeric screening by the Multiplex Ligation Dependent Probe Amplification (MLPA) Technique in 100 selected patients with idiopathic mental retardation (IMR) in Iran.

**Materials and Methods:**

A number of 100 patients with IMR, normal karyotypes and negative fragile-X and metabolic tests were screened for subtelomeric abnormalities using MLPA technique.

**Results:**

Nine of 100 patients showed subtelomeric abnormalities with at least one of the two MLPA kits. Deletion in a single region was found in 3 patients, and in two different subtelomeric regions in 1 patient. Duplication was only single and was present in 2 patients. Three patients were found to have both a deletion and duplication.MLPA testing in the parental samples of 7 patients which was accessible showed that 4 patients were de novo, 2 patients had inherited from a clinically normal mother, and one had inherited from a clinically normal father. Screening with the two MLPA kits (SALSA P036 and SALSA P070) proved abnormality in only five of the 9 patients.

**Conclusions:**

So, the prevalence rate of abnormal subtelomeres using MLPA technique in patients with idiopathic MR in our study was 5 - 9%, the higher limit referring to the positive results of one of the two MLPA kits, and the lower limit representing the results of positive double-checking with the two MLPA kits.

## 1. Background

Mental retardation/Developmental delay (MR/DD) is present in 1 - 3% of the general population (1, 2). MR is defined as a significant impairment of both cognitive (IQ < 70) and social adaptive functions, with onset before 18 years of age. MR is usually diagnosed in children older than 4 years. For younger children however, the term “developmental delay” is usually used.

The cause of MR is known in 50% of patients (3), and 25 - 50% of moderate to profound MR/DD is thought to have genetic etiology (4). An important cause has been shown to be chromosome abnormalities. Cytogenetically detectable and submicroscopic chromosomal rearrangements account for nearly 25% of all patients (5-7). It has been established that cryptic subtelomeric chromosomal imbalances can be present in 5 - 20 percent of patients with idiopathic mental retardation (IMR) (8-13). These abnormalities are less than 4 Mbp and cannot be detected using conventional cytogenetic chromosome banding resolutions. The subtelomeric regions are gene rich and very dynamic and heterogeneous. They are unique to each chromosome. The subtelomeric regions are rich in pseudogenes and repeat sequences which share homology between non homologous chromosomes resulting in mis-pairing at early meiotic prophase. This encourages increased recombination rate (14-17). However, increased level of nonhomologue telomere pairing can lead to recombination and gene dosage imbalance. Flint et al. (10) first reported subtelomeric screening for individuals with IMR. They reported an abnormality rate of 6% using FISH technique. Rooms et al. (12), using MLPA technique, reported the subtelomeric abnormality rate of 5.3% in 75 patients with IMR with or without dysmorphism. Some other authors have reported 6.7%, 4.4%, and 12.2% subtelomeric abnormality rates (18-20).

## 2. Objectives

In this study we reported the results of subtelomeric screening by MLPA technique in 100 selected patients with idiopathic MR and 50 normal control individuals.

## 3. Patients and Methods

### 3.1. Patient Data

A number of 100 patients (or their blood samples) were referred to the Genetics Research Center at the University of Social Welfare and Rehabilitation Sciences in Tehran, upon request. All patients had idiopathic mental retardation (IMR). Clinicians had not arrived at a specific clinical diagnosis and all patients had normal karyotypes findings using standard GTG high resolution banding technique and also negative results for fragile-X and metabolic tests. The intelligence quotient (IQ) was < 75 in all patients, which was assessed by psychologists mostly using the Raven IQ test. The age range of patients was 4 - 42 years, with an average age of 14.82, among which 73 patients (73%) were under 18 years old. Full clinical evaluation by pediatricians was accessible for 50 patients.

The control group consisted of normal healthy adult volunteers with no family history of MR. Parents were available for most abnormal patients, with whom the origin of abnormality was ascertained whenever necessary. The ethical codes of practice, as recommended by the ethical committee of the University of Social Welfare and Rehabilitation Sciences, were followed and the committee approved the research.

### 3.2. MLPA for Screening of Subtelomeric Regions

The kits used were SALSA P070 and P036 human telomere test kits (MRC-Holland, Amsterdam, Netherlands: http://www.mrc-holland.com/). The MLPA mix contained probes for all subtelomeric regions except the short arms of the acrocentric chromosomes (13p, 14p, 15p, 21p, and 22p). MLPA analysis was performed as suggested by the manufacturer. PCR amplification products were identified and quantified by capillary electrophoresis using ABI 3100 genetic analyzer. The fluorescent signal strength of the PCR products was determined using coffalyser software. For each person (patient and healthy normal individual) the normalized peak pattern of each subtelomeric region was divided by the average peak pattern of all the samples in the same experiment. The resulting values were approximately 1.0 for normal peaks, <0.75 for deletions, and > 1.3 for duplications. We were able to assess 50 patients using both SALSA P036 and SALSA P070 kits. Other patients were assessed only by the SALSA P036 kit. Abnormal patients found in this latter group were double checked by the SALSA P070 kit. The control group was assessed using the SALSA P036 kit.

## 4. Results

Full clinical evaluation by pediatricians was only accessible for 50 patients. Table 1 demonstrates the prevalence rate of some of the more common phenotypic abnormalities as well as some maternal or neonatal medical risk factors in those 50 patients. The most prevalent clinical findings were seizure (48%), growth failure (38%), microcephaly (30%), and facial abnormalities (20%), respectively. 

**Table1.** The Prevalence Rate of Some Common Phenotypic Abnormalities and Maternal or Neonatal Medical Risk Factors in 50 Patients With IMR for Whom Clinical Evaluation Was Possible

Type of Clinical Feature	Prevalence rate No., (%)
**Male gender**	31 (62%)
**Seizure**	24 (48%)
**Autistic Features**	9 (18%)
**Facial Dysmorphism**	11 (22%)
**Non facial Dysmorphism or other congenital anomalies**	10 (20%)
**Microcephaly**	15 (30%)
**Macrocephaly**	3 (6%)
**Low Birth Weight**	9 (18%)
**Growth Failure**	19 (38%)
**Maternal Miscarriages**	8 (16%)

Two of the patients who had originally been reported to have a normal karyotype by other laboratories were included in our MLPA testing procedure. However, MLPA revealed unbalanced chromosomes in the patients and with further investigation, the karyotypes of both patients proved to have abnormal findings, which in both patients had been inherited from a balanced maternal translocation. The karyotype, and MLPA results for these patients are presented in Table 2. Obviously, these two patients were consequently excluded from the project. 

**Table 2.** Karyotype, and MLPA Results for Two Patients Excluded From the Project

No	Patient Code	Karyotype	MLPA
**1**	1241	46, XY, der(13) t(7;13) (q32;q32) mat	46, XY
			mlpa (p036E1,p070B1)
			13qsubtel x1
			7qsubtel x3
**2**	34340	46, XY, der(18) t(6;18) (q25.3;q21.3) mat	46,XY
			mlpa(p036E1, p070B1)
			18qsubtel x1
			6qsubtel x3

In total, 9 of 100 patients showed subtelomeric abnormalities with at least one of the MLPA kits (Table 3). Some demographic characteristics, maternal and neonatal risk factors, and major clinical features of the 9 abnormal patients, as much as accessible, are demonstrated in Table 4. Deletion in a single region was found in 3 patients (3pter del, 2qter del, 15q11 del). Deletions in two different subtelomeric regions were identified in one patient (2pterdel+3pter del). Duplication was only single and was present in 2 patients (19qter dup, X/Yp dup). Three patients were found to have both a deletion and duplication (1pterdel+8qter dup, 10qter del+6pter dup, 22qterdel+19pter dup). 

**Table 3.** MLPA Results, Inheritance, and the Genes Involved for the 9 Patients With Abnormal MLPA Results With One or Two Kits

Patient Code	MLPA abnormality	Loss (Del)	Gain (Dup)	Inheritance	Involved genes	Function of involved genes	Reported as CNV^b^	
	Kit P036	Kit P070						
**29660**	46,XY,	46,XY,	3p		Mat^a^	CHL1	neural recognition molecule	Yes
	**mlpa(p036E1)**	mlpa(p070B1)						
	**3psubtel x1**	3psubtel x1						
**34580**	46,XX, mlpa(p036E1) 1psubtel x1	46,XX,	1p	8q	De novo	1p:TNFRSF4, TNFRSF18	TNFRSF: Apoptosis regulation	1p :
		**mlpa(p070B1)**						TNFRSF4 Yes
		**1psubtel x1**						
		**8qsubtel x3**				8q:RECQL4	RECQL4: DNA helicase	TNFRSF18 Yes
								**8q Yes**
**30240**	46,XY, mlpa(p036E1) 15q11 x1	46,XY, mlpa(p070B1) 15q11 x1	15q11		De novo	MKRN3	MKRN3: E3 ubiquitin ligase	MKRN3 Yes
						**NDN**		NDN YES
**21920**	46,XY, mlpa(p036E1) 19qsubtel x3	46,XY, mlpa(p070A2) 19qsubtel x3		19q	ND^a^	BC-2	components of ESCRT-III (endosomal sorting complex required for transport III)	Yes
**3240**	46,XY, mlpa(p036E1) X/Ypsubtel x3	46,XY, mlpa(p070A2) X/Ypsubtel x3		X/Yp	Mat	SHOX	associated with idiopathic growth retardation	Yes
**1521**	46,XY, mlpa(p036E1) 2p,3psubtel x1	46,XY,	2p,3p		ND	2p:ACP1	ACP1: phosphotyrosine protein phosphatase	2p Yes
							**CHL1: neural recognition molecule**	
		**mlpa(p070A2)**						
						**3p:CHL1**		3p Yes
		**x2**						
**20370**	46,XY,	46,XY,	10q	6p	De novo	10q:PAO,ECHS1	PAO: polyamine oxidase	10q:
								**PAO Yes**
	**mlpa(p036E1)**							
		**mlpa(p070A2)**					ESHS1: mitochondrial fatty acid beta-oxidation	
	**10qsubtel x1,**							
								**ECHS1 Yes**
						**6p: IRF4**		
	**6psubtel x3**							
							**IRF4: transcription factor**	
		**x2**						
								6p Yes
**22830**	46,XY, mlpa(p036E1) 2qsubtel x1	46,XY, mlpa(p070A2) x2	2q		Pata	CAPN10	Calcium dependent cysteine proteases	Yes
**89215**	46,XY,	46,XX, mlpa (p070A2) 19psubtel x3, 22qsubtel x1	22q	19p	De novo	19p:PPAP2C	PPAP2C: phosphatidic acid phosphatase	19p Yes
							**ARSA: cerebroside sulfate sulfatase**	
						**22q:ARSA**		
	**mlpa(p036E1)**							
								22q Yes
	**x2**							

^b^ Checked with http://projects.tcag.ca/variation/

^a^ Abbreviations: Mat, Maternal; ND, Not Determined; Pat, Paternal

**Table 4.** Clinical Findings in 9 Patients With Abnormal MLPA Results With at Least One MLPA Kit

Patient ID	Gender	Age	Level of MR	Seizure	Autistic Features	Dysmorphism	Micro cephalic	Other abnormal clinical features	Low birth weight	Growth failure	family history
**29660**	M	7	Profound	+	-	Strabismus, microstomia, micrognathia, protruded ears, broad nasal bridge, palmar transverse crease,	+	-Hyperactivity, stereotypic movements, motor delay, speech delay, mildly spastic muscular tone	-	+	History of neonatal death in other offspring
**34580**	F	5	Mod	-	+	Microphthalmia, hypotelorism, mildly protruded mandible, mild symmetric overriding of toes, mildly protruded breasts	-	Mild hyperactivity, drooling, astigmatism, abnormal wide-base gait, low muscular tone, motor delay, speech delay,	-	-	Cleft palate in brother, schizophrenic father, speech delay in third and fourth degree paternal relatives
**30240**	M	8	Mod ^a^	+	-	-	-	Bursting laughter, stereotype hand movement, motor delay, albinism	-	-	-
**21920**	M	19	severe	-	-	-	-	-	-	-	History of MR in two siblings
**3240**	M	12	Severe	-	-	some facial dysmorphism	-	-Ataxia, pemphigus	-	-	Seven patients with MR in the family, history of seizures in family members
**1521**	M	32	Severe	-	-	Minor facial dysmorphism, deafness, hypergonadism	-	-	NA^a^	NA	-
**20370**	M	14	Mod	-	-	blepharoptosis, - phimosis	+	-	NA	NA	-
**22830**	M	16	Mod	-	-	-	-	-	NA	NA	Family history of MR
**89215**	F	9	Mild	-	-	-.	+	Neurodevelopmental delay , speech delay	NA	NA	-

^a^ Abbreviations: Mod, Moderate; NA, Not Available

Parental blood samples were not available for two patients with abnormal results. However, the MLPA test in the parental samples of the other 7 patients showed that 4 patients were apparently de novo, 2 patients had inherited from the mother, and one had inherited from the father, all of whom were apparently clinically normal.

The abnormalities were detected by both SALSA P036 and SALSA P070 kits in five of the 9 patients, although in one of the five patients, who showed simultaneous deletion and duplication in kit P070 (patient 34580), kit P036 only detected the deletion. In four other patients the abnormality was not confirmed by the second kit, as follows: the abnormalities for patients with the code numbers 1521, 20370, and 22830 were detected only with the kit p036E1; whereas, for patient 89215, only the kit p070A2 was informative (Table 3). 

So, the prevalence rate of abnormal subtelomeres using MLPA in idiopathic MR patients in our study was 9% referring to the results of one of the two MLPA kits, but cut down to 5% considering the double-checking results with both MLPA kits.

Table 5 compares the prevalence rates of some of the more common clinical features in the 5 patients with abnormal MLPA, with the 47 MLPA normal patients in whom clinical evaluation was possible. 

**Table 5.** Comparison of Prevalence Rates of Some Common Clinical Features in IMR Patients With Abnormal MLPA Results, and Patients With Other Forms of IMR for Whom Clinical Evaluation Was Possible

Type of Clinical Feature ^a^	Prevalence in 5 patients with abnormal MLPA (%)	Prevalence in 47 patients with normal MLPA results (%)
**Male gender**	4 (80%)	29 (61.7%)
**Seizure**	2 (40%)	22 (46.8%)
**Autistic Features**	1 (20%)	8 (17%)
**Facial Abnormalities**	3 (60%)	9 (19.15%)
**Non facial Dysmorphism or other congenital anomalies**	2 (66.6%)	8 (17%)
**Microcephaly**	1 (33.3%)	14 (29.79%)
**Macrocephaly**	0	3 (6.38%)
**Low Birth Weight**	0	9 (19.15%)
** Growth Failure**	1 (33.3%)	18 (38.3%)
**positive family history of relevant disorders**	4 (80%)	7 (14.89%)

^a^ Abbreviation: IMR, idiopathic mental retardation

## 5. Discussion

The reports on the chromosome abnormality rate in Iranian patients with mental retardation are limited. A study by Behjati et al. (21) reported the rate of chromosome abnormality in Iranian patients with idiopathic mental retardation with consanguineous parents as 1.24%, which is rather low. In this study the subtelomeric abnormalities are reported in some Iranian patients with idiopathic mental retardation.

The subtelomeric regions are gene rich and a hotspot for recombination. It is now established that the rearrangements in subtelomeric regions can be the causative factor in 5 - 20% of patients with idiopathic MR (9). Therefore it seems necessary to perform subtelomeric screening in patients with idiopathic MR in whom karyotyping, fragile X and metabolic tests have normal findings.

Our data is comparable to other reported studies. Wu et al. (22) in a study on 451 Chinese children with unexplained developmental delay/mental retardation using subtelomeric MLPA probes detected an abnormality rate of 5.1%. The subtelomeric abnormality rates in patients with developmental delay/or dysmorphic features using MLPA technique has been reported as 5.9% by Ahn JW et al. (23) and 6.7% by Koolen et al. (18). Palomares et al. (24) reported a subtelomeric abnormality rate of 10% using MLPA in 50 patients with idiopathic MR and dysmorphism.

Among the 9 patients with MLPA abnormality in one or two kits, we were able to assess the inheritance status in 7 patients. In three patients the abnormalities were inherited from one parent, such that in patient 1 (del 3p) and in patient 5 (dupX/Yp), the abnormalities were inherited from the mother, and in patient 8 the 2q- they were inherited from the father, who were all clinically normal. Therefore these abnormalities are most probably polymorphic and not pathogenic. Del3pter, dupX/Ypter, and del2qter have already been reported as polymorphic sites (http://projects.tcag.ca/variation). In fact the only abnormal MLPA subtelomere in this study which has not been previously reported as CNV, using this site, was CAPN10 gene on 22q (Table 2). In 4 of the 7 patients, the inheritance was de novo and not present in either parent. However, to establish whether this abnormality is an imbalance inherited from parental balanced rearrangement, future testing using fluorescence in situ hybridization (FISH) technique is pending. 

In the five patients whose MLPA findings were confirmed with two kits, the abnormalities in two of the patients were de novo (patients 34580 and 302400), in two others (patients 29660 and 3240) the abnormality was maternally inherited, and in one it was nondetermined.

In the 5 tested patients for whom MLPA had abnormal results, some clinical symptoms or signs were exclusively present in only one of the 5 patients. This was not the case with the following clinical features: seizures (present in 2 patients), dysmorphism (facial dysmorphism detected in 3 and other forms of dysmorphism found in 2 patients), hyperactivity (present in 2), stereotypic movements (present in 2), motor delay (present in 2), speech delay (present in 2), and positive family history of relevant disorders (present in 4). Four of the 5 patients were male. Evidently, considering the small number of patients proven to be abnormal in MLPA testing, comparison of clinical features with those with normal MLPA is not a reliable one. However, it seems that all clinical features listed in Table 5 have higher prevalence rates in the former group as compared to the latter group, except for seizures, macrocephaly, low birth weight, and growth failure which seem to be less common in MLPA abnormal patients than other forms of idiopathic MR. 

In total, 9% of the patients showed subtelomeric abnormalities with at least one MLPA subtelomeric kit, 5% of which were detected by the two MLPA kits. Notably, two patients who had been mistakenly reported as having normal karyotypes results by other laboratories were shown to have chromosome imbalances by MLPA testing and were subsequently rekaryotyped, so we recommend the use of subtelomeric screening as a first-line testing approach for chromosome imbalances in patients with idiopathic mental retardation.
